# A Longitudinal Assessment of Diet Quality and Risks Associated with Malnutrition in Socioeconomic and Racially Diverse Adults

**DOI:** 10.3390/nu11092046

**Published:** 2019-09-02

**Authors:** Marie Fanelli Kuczmarski, Emily Stave Shupe, Ryan T. Pohlig, Rita Rawal, Alan B. Zonderman, Michele K. Evans

**Affiliations:** 1 Department of Behavioral Health and Nutrition, University of Delaware, 206C McDowell Hall, Newark, DE 19716, USA; 2 College of Health Sciences, University of Delaware, STAR, Newark, DE 19716, USA; 3 Department of Medical Laboratory Sciences, University of Delaware, 206C McDowell Hall, Newark, DE 19716, USA; 4 National Institute on Aging, Laboratory of Epidemiology and Population Sciences, NIH, 251 Bayview Blvd, Baltimore, MD 21224, USA

**Keywords:** diet quality, malnutrition, African American, MNA

## Abstract

Little is known about the effects of diet quality through adulthood and its association with malnutrition later in life. The first research objective was to evaluate diet quality assessed by Mean Adequacy Ratio (MAR) of United States African American and White adults (*n* = 2066), examined at baseline and two follow-up waves in the Healthy Aging in Neighborhoods of Diversity across the Life Span (HANDLS) study. The sample was split into cohorts by age at study baseline: Younger, <50, and older, ≥50 years. The second objective was to assess the association of MAR and risk for malnutrition in adults who were ≥60 years at wave 4 (*n* = 746). The Mini Nutritional Assessment was used to determine risk for malnutrition. At each of the three study waves, 17 micronutrients from two 24 h dietary recalls were used to calculate MAR. Over 13 years MAR changed minimally in the younger cohort as they aged from early to middle adulthood. In contrast, a statistically significant decline in MAR was observed for the older cohort between baseline (2004–2009) and wave 4 (2013–2017), with a greater degree of worsening at low energy levels. The risk for malnutrition was significantly associated with consuming a diet low in energy, lower protein as a percent of energy at baseline, as well as being food insecure, a current smoker, and having income <125% poverty. The risk for malnutrition was not associated with a change in protein intake in years prior to age 60, change in MAR scores across waves, MAR at wave 4, age, sex, race, or having hypertension or diabetes. These longitudinal study findings revealed that diet quality was not predictive of risk for malnutrition.

## 1. Introduction

Consuming a healthful diet throughout the life span reduces the risk of developing chronic diseases and malnutrition in all its forms [1]. A diet of high quality would be one that closely conforms to such healthful eating patterns as the Mediterranean diet, Dietary Approaches to Stop Hypertension (DASH) or Healthy Eating Index (HEI) [2]. The concept of diet quality is multidimensional [3], resulting in no single measurement approach. Diet quality has been measured by a variety of indices including but not limited to the HEI, dietary diversity indices, and Mean Adequacy Ratio (MAR).

Diet quality in this study was measured by MAR, an indicator of overall nutritional adequacy of a population based on an individual’s diet using the Dietary Reference Intakes for selected micronutrients [4]. Nutritional adequacy is defined as the intake of essential nutrients, needed to fulfill requirements for optimal health. While the MAR does not capture issues related to energy under- or over-consumption, its strength is the ability to provide information on the adequacy of an individual’s diet which can be paired with health measures [5].

While the diet quality of Americans is improving [6,7], it is still inadequate. Of public health concern are nutrients that are under-consumed and have direct health implications including calcium, potassium, dietary fiber, and vitamin D [2,8,9,10,11,12]. Overall diet quality of United States (US) adults aged 18–64 years is lower than that of older adults, aged 65 years and older, evidenced by a mean HEI-2010 score of 58.27 compared to 68.29 out of 100, respectively [13]. Diet quality is also higher in populations with higher socioeconomic status [7,14]. Regardless of age or income, improvements are still needed to comply with dietary guidance [15].

Adults over the age of 65 comprise the fastest-growing segment of the US population [16]. Compared to middle-aged adults, older adults can be at higher risk for poor nutritional status [17]. A decline in nutritional health may be due to several factors, including but not limited to inadequate energy and/or nutrient intakes, compromised nutrient metabolism, drug-nutrient interactions, altered nutrient needs, cognitive and physical decline, and socioeconomic changes [17,18,19,20,21]. Malnutrition in older adults in the community is often attributed to chronic inadequate energy and nutrient intakes [22,23,24]. The European Society for Clinical Nutrition and Metabolism defines malnutrition as “a state resulting from lack of uptake or intake of nutrition causing altered body composition (decreased fat-free mass and body cell mass), leading to diminished physical and mental function and impaired outcome from disease.” [25]. Protein-energy malnutrition is defined as the progressive loss of both lean body mass and adipose tissue resulting from insufficient consumption of protein and energy [26].

The prevalence of malnutrition in North America and Europe range from 1% to 15% in noninstitutionalized older adults [20,27,28]. Malnutrition among older adults is associated with increased morbidity and mortality and decreased quality of life [29,30]. Limited research has examined diet quality longitudinally throughout adulthood and risk for malnutrition in later years. Diets of high quality may be expected to lessen the risk of malnutrition and reduce the risk for the development of chronic conditions with aging [29,30,31,32,33]. Hengeveld and colleagues reported that poor diet quality, assessed by the HEI, was not a risk factor for the long-term development of protein-energy malnutrition in older adults who participated in the Health, Aging, and Body Composition study. Yet lower protein intake was associated with higher risk [34].

The effects of diet quality on health outcomes throughout the adult life span, starting around age 30 years, have not been thoroughly studied. It is not clear whether diet quality in young adulthood remains the same over middle and older adulthood in an urban population of low socioeconomic status and if there is an association of diet quality in middle-aged adults with risk for malnutrition in later years. The first study aim was to compare diet quality determined by the Mean Adequacy Ratio (MAR) of a younger and older cohort of African-American (AA) and White (W) participants examined at baseline and two follow-up waves in the Healthy Aging in Neighborhoods of Diversity across the Life Span (HANDLS) study. The second aim was focused on adults 60 years of age and older at wave 4 and was designed to assess the association of diet quality and risk for malnutrition determined by the Mini Nutritional Assessment (MNA), a validated screening tool for the detection of malnutrition [35,36].

## 2. Materials and Methods

### 2.1. Background on Healthy Aging in Neighborhoods of Diversity across the Life Span (HANDLS) Study

In 2004, the longitudinal HANDLS study was initiated. The objectives and 4-way cross factorial design of this study have been described elsewhere [37,38]. The 4 factors were age, race, sex, and poverty status. To date, 4 waves have been completed of which 3 were in-person examinations, namely baseline wave (2004–2009), wave 3 (2009–2013), and wave 4 (2013–2017); wave 5 began in 2017 and is ongoing.

At each wave, a battery of exams and interviews were conducted on Mobile Research Vehicles (MRVs). For the baseline wave, the other session was an in-home interview prior to their visit on the MRVs. For waves 3 and 4, a telephone interview was conducted after the visit to the MRVs. The data collected from participants include but were not limited to demographic information, a medical examination, two 24 h dietary recalls, cognitive evaluation, physical examination, bone density, muscle strength assessments, literacy testing, and a fasting blood draw. A complete listing of HANDLS variables by wave can be found elsewhere [38].

Human Institutional Review Boards at MedStar Health Research Institute, the National Institutes of Environmental Health Sciences, National Institutes of Health, and the University of Delaware approved the study protocol. All HANDLS participants provided written informed consent following their review of a protocol booklet in layman’s terms and watching a video describing all procedures. Participants received monetary compensation for participation.

### 2.2. Dietary Methods and Quality

**Figure 1:**
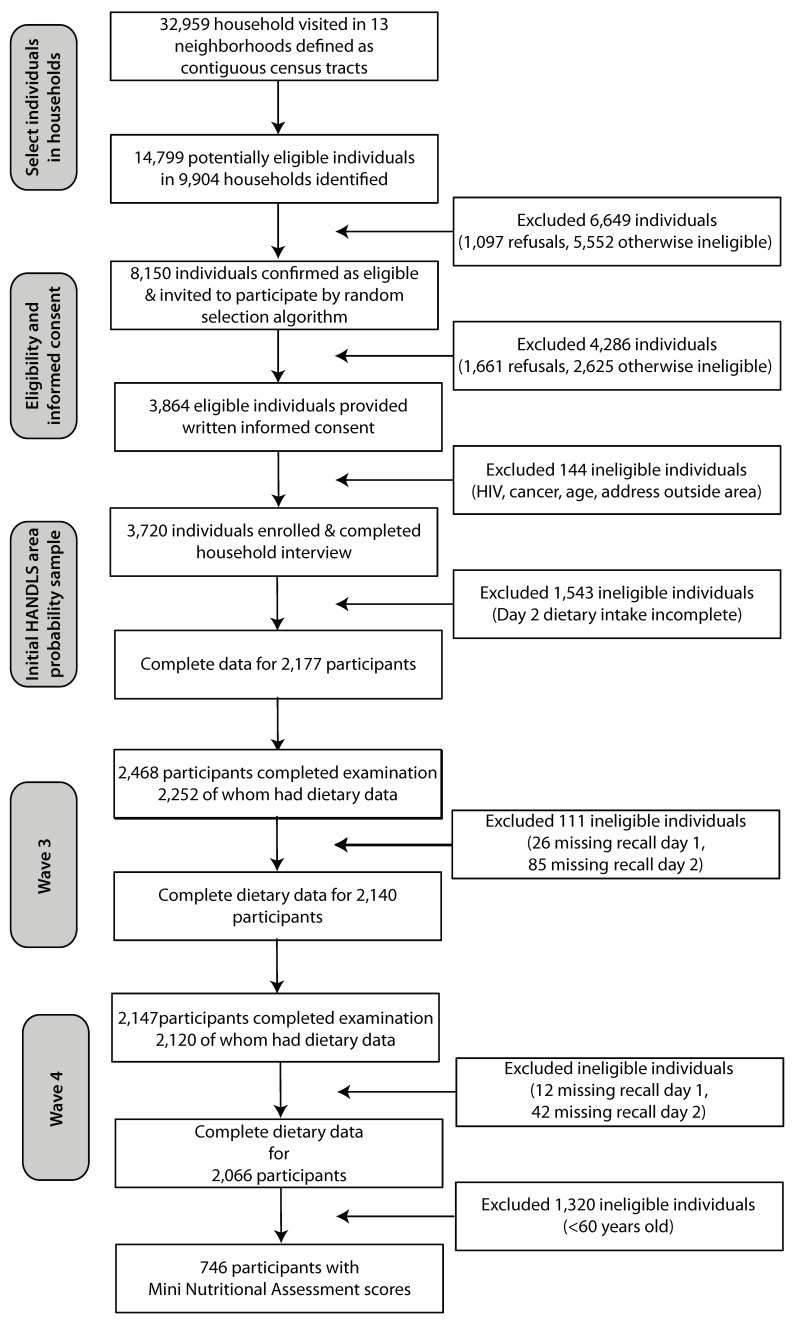
Participants completing two dietary recalls at each HANDLS study wave and participants with Mini Nutritional Assessment Scores. HANDLS—Healthy Aging in Neighborhoods of Diversity across the Life Span.

All 24 h dietary recalls were collected using the United States Department of Agriculture (USDA) 5-step Automated Multiple-Pass Method (AMPM) [39,40]. To assist participants with the recall of all foods and beverages and the estimation of quantities eaten, several cues and prompts during the administration of the AMPM, along with an illustrated food model booklet, measuring spoons and cups, and a ruler, were employed. Trained dietary interviewers conducted all the 24 h dietary recalls. The time between recalls ranged from approximately 4 to 10 days. Each food and beverage was coded using the USDA Survey Net data processing system, matching the foods with codes in the Food and Nutrient Database for Dietary Studies [41]. Of the 3720 participants examined in the baseline study, 2177 individuals completed 2 24 h dietary recalls; at wave 3, 2140 participants, and at wave 4, 2066 participants completed both 24 h dietary recalls (See Figure 1).

Nutrient Adequacy Ratio (NAR) for 17 micronutrients and MAR scores were based on food intakes only and used to assess diet quality [42,43]. The equations used to calculate NAR and MAR scores have been described elsewhere [43,44]. For each daily intake, the NAR and MAR were calculated and then averaged over the 2 days. The MAR score represents the nutrient-based diet quality variable with a maximum score of 100.

### 2.3. Malnutrition Evaluation

Malnutrition was assessed using the MNA-short form [35,45]. The MNA Short Form includes 6 of the original 18 items from the validated MNA. The 6 areas cover food intake, weight loss, mobility, psychological stress, neuropsychological problems, and Body Mass Index (BMI). The MNA is used for nutritional screening and is useful for identifying frail and malnourished older adults [36,46,47]. A score ≥12 out of 14 points indicated a normal nutritional status; a score between 8–11 points was considered at-risk for malnutrition and 0–7 points suggested malnutrition [48]. The risk of malnutrition detected by the MNA is before a severe change in body weight or serum proteins occurs.

### 2.4. Demographic and Health-Related Measures

Demographic characteristics measured included age (years), sex, race, poverty status, education, and smoking status. For this study, age at baseline was categorized as <50 years (referred to as the younger cohort and early to middle adulthood) and ≥50 years (referred to as the older cohort and middle to older adulthood). Race was self-reported only at baseline as AA or W. Similarly, income was self-reported only at baseline as household income either <125% or >125% of the 2004 Health and Human Services poverty guidelines; hereafter termed “poverty” [49]. Sex (male or female) was only also reported at baseline with the exception of those persons who changed genders. Education was categorized as <12th grade education or ≥12th grade education/general equivalency diploma based on questions asked at baseline and wave 3. Cigarette smoking was coded as current smoker or non-smoker at each wave. For each wave, food insecurity was defined as a positive response to one question, “In the past 12 months, did you ever eat less than you felt you should because there was not enough money to buy food”?

BMI was calculated as the ratio of measured weight (kg) to height (m) squared at each wave. Weight and height were measured using a calibrated Med-weigh, model 2500 digital scale, and a height meter (Novel Products, Inc., Rockton, IL, USA), respectively. The presence of diabetes and hypertension was assessed either by self-report, medication use, or blood glucose/blood pressure values. Diabetes was defined as ≥126 mg/dL [50]. In the HANDLS study, hypertension was defined as systolic blood pressure ≥140 mm Hg or diastolic blood pressure ≥90 mm Hg [51]. For consistency across waves of data, these values were used at each wave with recognition of the 2017 guidelines for high blood pressure [52].

Fasting venous blood specimens were collected from participants during their MRV visit and analyzed at the Nichols Institute of Quest Diagnostics, Inc. (Chantilly, VA, USA). Fasting blood results utilized for the present study included serum measures of albumin (g/L), magnesium (mg/dL), potassium (mmol/L), ferritin (ng/mL), 25-hydroxy(OH) Vitamin D (ng/mL), total cholesterol (mg/dL), triglycerides (mg/dL), white blood cell (WBC) count (count*10^9^/L), platelet count (10^9^/L), and albumin/globulin ratio (g/dL). Serum albumin and magnesium were measured by the standard clinical laboratory spectrophotometric assay. Albumin/globulin ratio was calculated using albumin and globulin, calculated as total protein-albumin. Potassium was assessed using an ion-selective electrode. Serum ferritin was measured using a standard chemiluminescence immunoassay. Serum 25-hydroxy (OH) Vitamin D was measured using an immunoassay. Total serum cholesterol and triglycerides were assessed using a spectrophotometer (Olympus 5400, Olympus, Melville, NY, USA). High-sensitivity C-Reactive Protein (hs-CRP) levels (mg/L) were assessed by the nephelometric method utilizing latex particles coated with CRP monoclonal antibodies. WBC count and platelet count were assessed using the microscopy method.

### 2.5. Statistical Analysis

Means and standard errors for continuous variables and the proportion of participants for relevant categorical variables were calculated. Analysis of variance (ANOVA) was used to compare demographic and lifestyle factors, diet quality, and physical performance measures between baseline and wave 3, baseline and wave 4, and waves 3 and 4 by age cohorts (younger, older) and between normal nutritional status and at-risk for malnutrition categories. For sample characteristics categorical data, χ^2^ tests were used. Statistical significance was established at *p* < 0.05, *p*-values were adjusted for multiple comparisons of continuous variables using the Bonferroni test. All statistical analyses were performed with IBM SPSS Statistics for Windows v25 (2017; IBM Corporation, Armonk, NY, USA) and R v3.5.3 (2019; R Foundation for Statistical Computing, Vienna, Austria).

To examine the change in MAR over time, a linear mixed-effects model was used. The model included age group (dichotomized as <50 years and individuals ≥50 years at baseline), sex, race, poverty, smoking status, education (dichotomized to those with at least a high school or general education development (GED) equivalency and those without), and energy as predictors. The model also included the 6-way interaction of age, age group, sex, race, poverty, and energy as well as all the lower order interactions within. Cigarette smoking and educational attainment were included as main effects only. Although there was not a significant 6-way interaction, there were some significant 5-way interactions. For brevity, only significant effects based on the 5-way interactions will be discussed in the results section. Full model results are in Supplementary Table S1.

As a subsequent analysis to explore variables associated with wave 4 MNA scores, sequential linear multivariable regression was performed. The first block included sex, race, poverty, hypertension, diabetes, smoking, age, energy, and food insecurity. Sex, race, and poverty were defined at baseline while hypertension, diabetes, smoking, age, energy, and food insecurity were defined at Wave 4. In the second block, protein percent (percent of the total energy that was protein) at baseline and the change in protein from baseline were included. Protein as a percentage of energy was significant but change in protein intake across waves was not. In the final block, indices of diet quality, namely the average MAR score at wave 4 and the change in MAR were added. The second block significantly improved the model, protein percent was significant, but the third block did not. Since neither MAR variable was significant, the model reported in Supplementary Table S2 is from the second block.

## 3. Results

### 3.1. Population Characteristics

Demographic and health characteristics of HANDLS study participants <50 years and individuals ≥50 years at baseline, by study wave, are provided in Table 1. Given the study design, similar percentages for men and AA were seen for both age cohorts. Approximately 60% of both age cohorts were AA, and 40% were men. Among the younger cohort, about 45% had incomes <125% poverty across all study waves. Among the older cohort, there was a significant difference in the percentage of the sample with incomes ˂125% poverty at baseline (40%) compared to waves 3 and 4 (35%). Between 34 to 40% of the study sample had ˂12 years of education (Table 1).

In the younger cohort, approximately half of the sample smoked, with 48% being the lowest percentage, reported at wave 3. While the percentage of hypertensive individuals increased over the life span (from 31.5% at baseline to 55.5% at wave 4), the percentage of persons with diabetes was similar at baseline and wave 3 (~11–13%) but then significantly rose at wave 4 to about 20%. Mean BMI increased significantly between baseline and wave 3 and then plateaued (Table 1). From baseline to wave 3, there was a shift in BMI classification with significantly fewer participants (32.9% to 25.8%) categorized as having a healthy BMI (<25 kg/m^2^) and significantly more classified as obese (≥30 kg/m^2^) (39.7% to 47.6%) (data not shown).

In the older cohort, the percent of current smokers decreased from baseline to wave 3 (42.2% to 33.7%) and then remained the same at wave 4. Participants with diabetes increased over the three study waves from approximately 23% to 31%. With each subsequent wave, the percent with hypertension increased (Table 1). At wave 4, 77.5% were hypertensive. Significantly more participants were obese at wave 4 (49.4%) compared to baseline (44.8%). As anticipated, BMI increased over the life span until around age 60 years and then declined. The ≥50-year age group had lower BMI compared to the <50-year age group (Table 1).

### 3.2. Comparison of Longitudinal Diet Quality of Younger and Older Cohorts

This study found that across the three study waves (~13 years), diet quality remained consistent as evidenced by a change in MAR score from baseline to wave 4 of approximately a 2.2% in the younger cohort and about 2.6% in the older cohort. Like the lack of significant change in micronutrient quality of the diet documented by the MAR scores in the younger cohort, energy intake and the percentage of participants consuming less than the Recommended Dietary Allowance (RDA) of 0.8 gm protein per kg body weight remained similar over time (Table 1). For the older cohort, energy intake also appeared unchanged over time. However, in wave 4, significantly more of the older cohort consumed less than the recommended protein intake compared to baseline (55.5% vs. 49.3%) (Table 1).

MAR scores were higher for women than men (*p* < 0.001), and for those who completed high school (*p* < 0.001) at each wave. At the 2000 kcal intake, the approximate mean energy intake of the HANDLS study sample, the MAR scores of persons in the older cohort were significantly higher only at baseline (*p* < 0.001) and wave 3 (*p* = 0.001) compared to those scores for persons in the younger cohort. The MAR scores of persons who were food secure decreased significantly from baseline 80.39 ± 0.56 to Wave 3 78.59 ± 0.46 (*p* = 0.005) and Wave 4 78.39 ± 0.45 (*p* = 0.002). Across waves, the MAR scores of persons experiencing food insecurity remain unchanged (75.84 ± 1.27 to 77.48 ± 0.65, *p* = 0.212) (data not shown).

The NAR scores for the 17 nutrients used to calculate MAR are provided in Supplementary Table S1. For seven of the 17 nutrients, there was no change over time for persons in the younger cohort. NAR scores of calcium, magnesium, iron, and vitamins D and E significantly increased, while scores significantly decreased for copper, zinc, and vitamin A. Among persons in the older cohort, NAR scores for eight nutrients, namely calcium, copper, iron, zinc, and vitamins A, B_6_, B_12_, and C, significantly decreased over time while only the NAR score for vitamin E significantly increased. The remaining NAR scores were not significantly different from baseline to wave 4.

**Figure 2:**
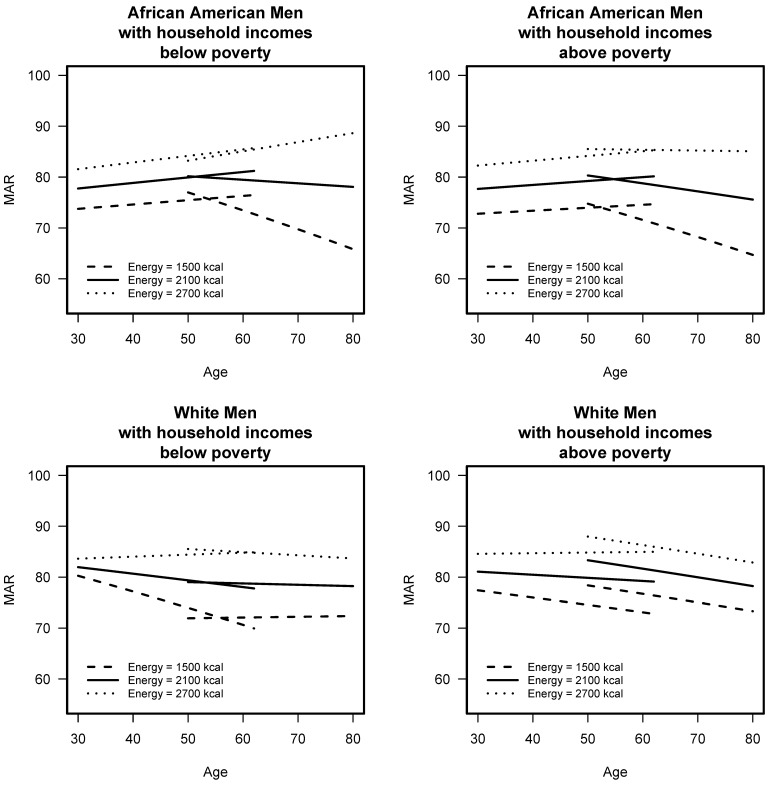
Mean Adequacy Ratio (MAR) scores across baseline and waves 3 and 4 of HANDLS study for men by initial age cohort (<50 or ≥50 years at baseline,) and by poverty (<125% or >125% poverty threshold) for three levels of energy intake. HANDLS-Healthy Aging in Neighborhoods of Diversity across the Life Span.

**Figure 3:**
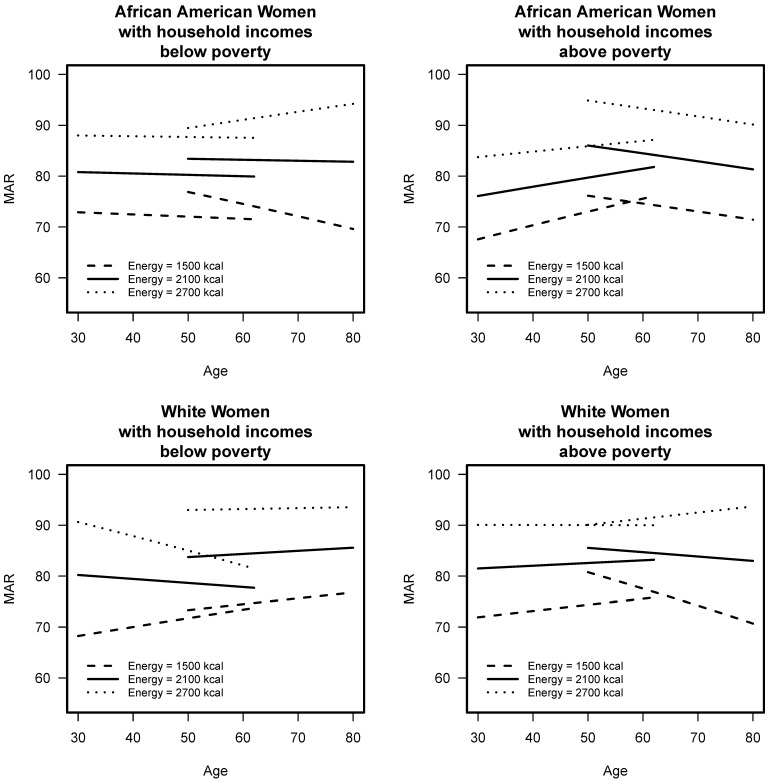
Mean Adequacy Ratio (MAR) scores across baseline and waves 3 and 4 of HANDLS study for women by initial age cohort (<50 or ≥50 years at baseline,) and by poverty (<125% or >125% poverty threshold) for three levels of energy intake. HANDLS-Healthy Aging in Neighborhoods of Diversity across the Life Span.

To assess change over time, the mixed models results show that diet quality was influenced by age, sex, race, poverty, education, energy, cigarette smoking, and three 5-way interactions (Supplementary Table S2). The interaction of age group by sex by race by poverty by energy is visually depicted for men in Figure 2 and for women in Figure 3. Diet quality, assessed by MAR scores, is shown at energy levels of 1500, 2100, and 2700 kcal, levels, which coincide with the energy distribution of a majority of HANDLS study sample. Each line represents MAR over time from baseline to wave 4.

For AA men with lower household incomes (<125% poverty), MAR in the younger cohort was stable over time, slightly increasing, and for the older cohort, MAR scores worsened over time, for all energy intake levels with a greater degree of worsening seen in the lowest energy intake level. For AA men with higher household incomes (>125% poverty), MAR in the younger cohort was stable over time, slightly increasing. For the older cohort, MAR scores worsened over time for low and moderate energy intake levels with a greater degree of worsening seen in the lowest energy intake level, and those in the highest intakes actually saw a small increase in MAR scores. For W men with lower household incomes, MAR in the younger cohort was stable over time for those with higher energy intake levels and got worse for those with moderate and low energy intake levels. For older W men with lower incomes, MAR scores were consistent over time, for all energy intake levels. For W with higher household incomes, MAR in the younger cohort was stable over time for all energy intake levels, and for the older cohort MAR, scores got worse over time for all energy intake levels.

For AA women with lower household incomes (<125% poverty), those who started in the younger cohort did not see a change in their MAR over time, regardless of their energy consumption level, whereas the older cohort saw a worsening MAR for those who consumed lower amounts of energy and those who consumed high amounts of energy saw an increase in their MAR. This finding was in contrast to AA women with higher household incomes (>125% poverty), which saw an increase in MAR for the younger cohort and a decrease in MAR for the older cohort; these trends were consistent across energy consumption levels. For W women with lower household incomes, the older cohort did not see much of a change in MAR over time regardless of energy level, but for the younger cohort, those with the highest energy intakes, they had a MAR that got worse over time, and those with the lowest intakes saw a slight increase in MAR over time. For W women with higher household incomes, those who started in the younger cohort did not see a change in their MAR over time, regardless of their energy consumption level, whereas the older cohort saw a declining MAR for those who consumed lower amounts of energy and those who consumed high amounts of energy saw an increase in their MAR.

### 3.3. Characteristics of Persons aged ≥60 years at Wave 4 At-Risk for Malnutrition

The average age of the sample who completed the MNA was 66 years. Table 2 provides a comparison of HANDLS study participants ≥60 years of age who were identified by the MNA as having normal nutritional status (*n* = 435) and those who were either at-risk for or were malnourished (*n* = 277 and *n* = 34, respectively). For purposes of simplicity, the latter group will be defined as “at-risk” in the results and discussion. Participants at-risk for malnutrition were more likely to have baseline incomes <125% poverty, have ˂12th grade education and smoke at wave 4 compared to persons with normal nutritional status. Although their BMI was significantly lower, the BMI of both groups indicated obesity. Hypertension but not diabetes was more prevalent among the at-risk group. Approximately 16% of persons in the at-risk group had CVD events between baseline and wave 4 compared to 9% of persons in the normal nutritional status group (*p* = 0.03). Additionally, persons at-risk were more likely to report food insecurity, were more likely to have symptoms of depression, as well as more frequently report not feeling like eating or having poor appetite due to depression at wave 4 compared to those persons defined as having normal nutritional status (Table 2). The prevalence of diagnosed depression, bipolar disorder, suicidal thoughts/attempts, and anxiety disorder at wave 4 was also significantly higher in these individuals at-risk (Table 2).

While there were no significant differences between wave 4 mean MAR scores, energy intake or percentage of energy from protein or fat, the at-risk group had significantly lower NAR scores for vitamin C, thiamin, riboflavin, niacin, folate, copper, magnesium, selenium, and zinc (9 of 17 nutrients) and significantly higher percent of their energy intake from carbohydrates and sugar compared to individuals categorized as having normal nutritional status. Examination of wave 4 blood values provided in Table 2 revealed that those at-risk had significantly lower serum albumin, albumin/globulin ratio, potassium, and vitamin D (Table 2) than those with normal nutritional status. There were no differences for hs-CRP levels, WBC count, platelet count, serum magnesium or triglycerides (Supplementary Table S3).

Compared to people considered to have normal nutritional status, those at-risk were more likely to report that they accomplished less due to health and that health limited their ability to climb stairs and perform moderate activities. They were also more likely to report their health as fair or poor. In addition, those at-risk were more likely to report difficulty walking ¼ mile, walking upstairs, standing up from a chair, and using their fingers (Table 2).

### 3.4. Association of Diet Quality with Malnutrition

Being food insecure, a current smoker, having income <125% poverty, and consuming a diet lower in energy and in the percent of energy as protein, were significantly associated with risk for malnutrition (Supplementary Table S4). Risk of malnutrition was not associated with age, sex, race, having hypertension or diabetes, or change in protein intake in years prior to age 60.

## 4. Discussion

This longitudinal study documented consistent diet quality in the younger cohort of a racially and socioeconomically diverse urban US sample as they aged from early to middle adulthood. A small yet statistically significant decline in diet quality was observed for the older cohort as they aged from middle to older adulthood. Batis and colleagues followed the diets of young and middle adults into older adulthood (age 18–65) and also found that diet remains relatively stable over time [53]. Like other studies with adults, the diet quality of older-aged groups was better compared to younger-aged adults [54]. The study findings were also similar to previous research, which reported lower education and food insecurity to be associated with lower diet quality [21,55].

While the interaction of race with poverty was associated with diet quality, only poverty, but not race, was associated with risk for malnutrition. Approximately 5% of the HANDLS study sample ≥60 years at wave 4 were categorized as malnourished and 37% as at-risk for malnutrition. Unlike Crichton and colleagues who found women at greater risk for malnutrition, sex was not associated with risk for malnutrition among participants of the HANDLS study [56].

Diet quality measured by MAR in years preceding the age of 60 years was not associated with risk for malnutrition in our independent community sample aged 60 years and older. However, lower energy and protein intakes were associated with risk for malnutrition for the HANDLS sample. Despite the use of a different diet quality measure, namely the HEI, Hengeveld and colleagues also reported that poor diet quality was not a risk factor for the development of protein-energy malnutrition in US community-dwelling AA and W adults 70–79 years of age [34].

The MAR score served as an indicator of the overall micronutrient adequacy of the diets consumed by the HANDLS sample. The lowest MAR scores were observed in wave 4 for those in the HANDLS older cohort. Their unadjusted mean score of 74 out of a maximum of 100 indicated that the diet was providing adequate amounts of most micronutrients. However, since the RDAs are defined as covering the required intake for all but 2–3% of the population, it is possible that nutrient deficiencies still exist. A closer examination of the NAR scores revealed that the micronutrients most likely to be under-consumed, regardless of age group, were calcium, magnesium, and vitamins A, C, E, and D. This finding was expected since the HANDLS study participants consume a Western diet pattern [43,57]. Others have documented that persons who consume a Western diet are less likely to achieve adequate intakes of vitamins A, C, and E, folate, magnesium, calcium, iron, and selenium [58,59]. These disparities in micronutrient intakes have been associated with the health inequalities observed across race/ethnic and sex groups [60].

Malnutrition in older adults is often the consequence of energy and protein deficiencies [29,30]. While energy requirements decrease with age due to altered body composition and a decrease in physical activity [31], protein requirements increase for muscle maintenance, immunity from disease, wound healing, and many other bodily functions [10,31]. For healthy older adults, it has been recommended that protein intakes should be 1.0 to 1.2 gm/kg body weight and for those who are malnourished or at-risk for malnutrition, intakes of 1.2 to 1.5 gm/kg body weight have been proposed [29]. Yet the mean protein intake of the HANDLS study participants who were 60 years of age and older at wave 4 was only about 0.82 gm/kg body weight. Low energy intake at wave 4 and low protein intake, expressed as a percentage of energy at baseline, were associated with risk for malnutrition in this study. Hengeveld et al. also reported that lower protein intake was associated with a higher risk for protein-energy malnutrition [34].

This study also confirmed the role of poverty as a risk factor for malnutrition in older adults [30,61]. HANDLS study participants at-risk for malnutrition were more likely to have baseline incomes ˂125% of the poverty level and were more likely to report food insecurity at wave 4. The financial strain associated with low income may impact the quality and quantity of food available to an individual as well as be a barrier to obtain and prepare meals [30,62,63]. In addition to poverty, being a current smoker was associated with increased risk for malnutrition among HANDLS study participants. This finding may be attributed to the association of smoking with oxidative stress, which may inhibit protein synthesis and increase the loss of muscle mass [64,65].

Like income, health, even perceived health, and physical functioning may affect the ability to perform daily activities and exacerbate problems associated with obtaining, preparing, and consuming adequate energy and nutrients [27,29,31]. The at-risk HANDLS study group was more likely to report their health as “fair” or “poor” compared to “excellent” or “good”. They had more difficulty with such physical performance measures as climbing stairs, standing up from a chair, and walking a ¼ mile and reported that their health limited moderate activities. These findings are supported by evidence that frailty and food insufficiency are associated [66].

Researchers have also documented the association between frailty and malnutrition and/or risk for malnutrition [28,67]. Deficits in protein may lead to loss of lean body mass and a decline in muscle strength and physical functioning [11,34]. Low serum vitamin D is associated with decreased muscle function, physical performance, and increased frailty and disability [11,68]. Nutrients such as vitamin C, potassium, and magnesium are associated with muscle strength and performance, and deficiencies of these nutrients increase the risk for frailty and falls [69,70]. Additionally, nutrients such as vitamin C, selenium, and zinc have antioxidant properties and protect against oxidative stress, which may be a factor contributing to poor muscle function [70,71]. The NAR scores for magnesium, selenium, zinc, and Vitamin C of HANDLS study participants at-risk for malnutrition were significantly lower than those with normal nutritional status.

The inflammatory process may also contribute to impaired physical function since it has been hypothesized to be a determinant of frailty [72]. Biomarkers for malnutrition and inflammation are low serum albumin and elevated hs-CRP [73]. Compared to individuals with normal nutrition status, the at-risk group had lower serum albumin levels, which may reflect not only protein deficiency, but also their consumption of low energy diets at wave 4. Although hs-CRP values did not differ between the groups, hs-CRP level was >3 mg/L in both groups, indicating inflammation. The lower albumin/globulin ratio adds further support to the presence of inflammation in the at-risk group [74]. A Western diet has been labeled as pro-inflammatory [75], thus it was not unexpected to find that there was no significant difference in hs-CRP for the HANDLS study at-risk and normal nutritional status groups. However, the percent of individuals with a CVD event since baseline in the at-risk group was twice that of the normal nutritional status group.

This study has many strengths, including the relatively large sample of diverse urban adults, the longitudinal design, and the use of multiple dietary recalls to determine the estimation of typical energy and nutrient intakes. The use of USDA AMPM has been shown to reduce bias in the collection of energy intakes and to provide accurate estimates of sodium intake [39]. Furthermore, the risk of malnutrition was assessed using the validated MNA, which is highly recommended as the basis for nutritional evaluation for older adults [35]. The authors decided to evaluate diet quality based on only food and beverage intakes. Some may view the exclusion of nutritional supplements as a limitation. Data on nutritional supplements were obtained from participants interviewed in waves 3 and 4. Incorporation of the nutrient intakes from supplements from about 40% of the sample who use supplements would increase their MAR scores. Other limitations include the limited number of nutritional biomarkers and inherent errors associated with the 24 h dietary recall [76].

## 5. Conclusions

To our knowledge, this is the first study to describe diet quality based on longitudinal dietary data for low-income urban AA and W individuals in the US. This study found that diet quality, assessed by MAR, did not change significantly in the younger cohort of the HANDLS study participants over a ~13-year period. However, for the older cohort, MAR scores decreased between baseline and wave 4 and intakes of inadequate protein increased at wave 4 of the study. While diet quality was not associated with risk for malnutrition, diets low in energy and protein, smoking, food insecurity, and low income (<125% poverty) were associated with increased risk of malnutrition in older adults over 60 years examined in wave 4 of the HANDLS study. The finding that diet quality was not associated with risk for malnutrition corroborates those of Hengeveld and colleagues who used data from the Health, Aging, and Body Composition study, which was comprised of 45% women and 33% AA, despite differences in diet methods and diet quality indices [34].

Nutrition education programs targeted at low-income families have been proven to be effective in improving their food resource management skills, enabling them to access healthier foods [77]. Education for older adults on safe storage and preparation of foods to improve their diet and nutritional status is recommended by professionals [29]. In summary, to reduce the risk for malnutrition in community-dwelling older adults, providing resources on nutrient-rich food selections on a limited budget would be beneficial.

## Figures and Tables

**Table 1:** Demographic and health characteristics and diet quality categorized by age at baseline of individuals examined in the HANDLS Study, 2004–2017.

Characteristics	Baseline Age <50 Years Younger Cohort			Baseline Age ≥50 Years Older Cohort		
	Baseline	Wave 3	Wave 4	Baseline	Wave 3	Wave 4
*Demographics*	*n* = 1194	*n* = 1190	*n* = 1198	*n* = 983	*n* = 950	*n* = 870
Age, X ± SE	41.34 ± 0.16 ^a^	46.60 ± 0.16 ^b^	50.38 ± 0.17 ^c^	57.00 ± 0.14 ^a^	61.41 ± 0.14 ^b^	65.24 ± 0.15 ^c^
African American, %	59.1 ^a^	63.4 ^a^	63.1 ^a^	56.4 ^a^	59.1 ^a^	57.8 ^a^
Men, %	43.1 ^a^	40.9 ^a^	41.7 ^a^	43.8 ^a^	41.5 ^a^	40.2 ^a^
Poverty, <125%, %	45.3 ^a^	43.4 ^a^	45.3 ^a^	40.0 ^a^	35.3 ^ab^	34.5 ^b^
Education, <12th grade, %	33.7 ^a^	39.6 ^a^	39.3 ^a^	35.0 ^a^	35.3 ^a^	34.0 ^a^
Smoker, %	53.3 ^a^	48.0 ^b^	54.7 ^c^	42.2 ^a^	33.7 ^b^	34.8 ^b^
*Health Conditions*						
Diabetes, %	11.4 ^a^	12.9 ^a^	19.8 ^b^	23.4 ^a^	25.9 ^ab^	30.7 ^b^
Hypertension, %	31.5 ^a^	42.2 ^b^	55.5 ^c^	63.9 ^a^	68.3 ^b^	77.5 ^c^
BMI, kg/m^2^, X ± SE	29.38 ± 0.23 ^a^ (*n* = 1192)	30.69 ± 0.24 ^b^ (*n* = 1188)	31.06 ± 0.24 ^b^ (*n* = 1190)	30.34 ± 0.24 ^a^ (*n* = 982)	30.74 ± 0.25 ^a^ (*n* = 948)	30.77 ± 0.26 ^a^ (*n* = 860)
*Diet-related Measures*						
MAR, X ± SE	78.30 ± 0.54 ^a^	77.49 ± 0.40 ^a^	77.90 ± 0.41 ^a^	77.68 ± 0.60 ^a^	76.93 ± 0.45 ^a^	74.24 ± 0.51 ^b^
Energy, kcal, X ± SE	2121 ± 30 ^a^	2151 ± 26 ^a^	2088 ± 25 ^a^	1867 ± 28 ^a^	1867 ± 25 ^a^	1796 ± 24 ^a^
Protein, <0.8 g/kg, %	44.0 ^a^ (*n* = 1192)	43.4 ^a^ (*n* = 1188)	48.0 ^a^ (*n* = 1190)	49.3 ^a^ (*n* = 982)	51.2 ^ab^ (*n* = 948)	55.5 ^b^ (*n* = 860)

Abbreviations: HANDLS—Healthy Aging in Neighborhoods of Diversity across the Life Span, SE- Standard Error, MAR—Mean Adequacy Ratio, BMI—Body Mass Index. Prediabetes = fasting glucose of 100–125 mg/dL; diabetes = fast glucose ≥126 mg/dL; hypertension ≥140 mm Hg for the systolic blood pressure measurement, or ≥90 mm Hg for the diastolic measurement. Superscripts with different letters in a row with each age group are significantly different *p* < 0.05.

**Table 2:** Demographics and diet- and health-related characteristics of HANDLS study participants aged ≥60 years at Wave 4 categorized by risk for malnutrition, screened by Mini Nutritional Assessment.

Characteristics	Normal Nutrition Status (*n* = 435)	At-Risk for Malnutrition (*n* = 311)	*p*-Value
*Demographics*			
Age, years, X ± SE	66.34 ± 0.19	65.81 ± 0.22	0.064
Men, %	38.9	41.3	0.492
African American, %	58.6	64.4	0.109
Poverty <125%, %	28.3	42.6	<0.001
Education, % <12th grade	27.4	39.0	0.001
Smoker, %	28.9	45.2	<0.001
*Health Conditions*			
BMI, kg/m^2^, X ± SE	31.42 ± 0.31	30.15 ± 0.50	0.025
CVD event since Baseline, %	8.7	15.6	0.030
Diabetes, %	21.9	24.6	0.622
Hypertension, %	59.9	69.5	0.007
Symptoms of depression, CES-D, %	26.4	41.2	<0.001
Diagnosed depression, %	26.0	44.2	<0.001
Diagnosed bipolar disorder, %	2.8	6.5	0.015
Diagnosed suicidal thoughts, %	4.8	11.0	0.002
Diagnosed anxiety disorder, %	13.8	27.7	<0.001
*Diet-related Measures*			
Food insecure, %	15.0	24.6	0.002
Not feel like eating, poor appetite most of the time, %	3.3	8.1	<0.001
Energy, kcal, X ± SE	1833 ± 32	1713 ± 40	0.017
MAR Wave 4, X ± SE	75.43 ± 0.70	72.69 ± 0.87	0.184
NAR Vitamin C, X ± SE	0.605 ± 0.017	0.553 ± 0.020	0.049
NAR Thiamin, X ± SE	0.887 ± 0.009	0.839 ± 0.012	0.001
NAR Riboflavin, X ± SE	0.938 ± 0.007	0.907 ± 0.009	0.005
NAR Niacin, X ± SE	0.946 ± 0.006	0.919 ± 0.009	0.011
NAR Folate, X ± SE	0.814 ± 0.011	0.775 ± 0.013	0.022
NAR Copper, X ± SE	0.881 ± 0.009	0.844 ± 0.011	0.007
NAR Magnesium, X ± SE	0.659 ± 0.010	0.607 ± 0.013	0.001
NAR Selenium, X ± SE	0.977 ± 0.004	0.960 ± 0.006	0.017
NAR Zinc, X ± SE	0.819 ± 0.010	0.775 ± 0.013	0.005
Protein, gm/kg, X ± SE	0.82 ± 0.02	0.83 ± 0.03	0.884
Protein, % energy	15.8 ± 0.2	15.5 ± 0.3	0.330
Carbohydrate, % energy	47.2 ± 0.5	48.7 ± 0.5	0.037
Sugar, % energy	21.8 ± 0.4	23.3 ± 0.5	0.022
Total fat, % energy	36.6 ± 0.4	35.7 ± 0.4	0.087
*Clinical and Physical Measures*			
Self-rated Health as poor/fair, %	18.4	31.3	<0.001
Accomplished less due to health, most of the time, %	8.2	16.9	<0.001
Health limits climbing stairs, a lot, %	12.8	25.6	<0.001
Health limits moderate activities, a lot, %	11.1	21.8	<0.001
Difficulty walking 1/4 mile; %	23.6	35.9	<0.001
Difficulty walking up 10 stairs; %	19.2	29.2	0.004
Difficulty standing up from chair; %	22.2	33.6	0.003
Difficulty carrying 20 lbs, %	13.4	20.3	0.068
Difficulty using fingers, %	17.8	26.6	0.004
*Biochemical Measures*			
Hs-CRP (mg/L), X ± SE	4.84 ± 0.42	6.07 ± 0.67	0.102
Albumin/Globulin ratio (g/dL); X ± SE	1.42 ± 0.01	1.35 ± 0.02	0.002
Albumin (g/dL), X ± SE	4.25 ± 0.02	4.18 ± 0.02	0.005
Potassium (mmol/L), X ± SE	4.24 ± 0.02	4.13 ± 0.03	0.003
Serum 25 OH Vitamin D (ng/mL), X ± SE	31.17 ± 0.75	28.12 ± 0.89	0.009

Abbreviations: HANDLS—Healthy Aging in Neighborhoods of Diversity across the Life Span, BMI—Body Mass Index, MAR—Mean Adequacy Ratio, NAR—Nutrient Adequacy Ratio, Hs-CRP—High sensitivity C-reactive protein. Defined by an affirmative response to the question, ‘Did you eat less because of insufficient money for food in the past month? Bolded font was used to emphasize *p*-values significant at <0.05 level.

## Supplementary Materials

The following are available online at https://www.mdpi.com/2072-6643/11/9/2046/s1, Table S1. MAR and NAR scores of HANDLS study sample at Baseline, wave 3 and wave 4, Table S2: Mixed effects regression with Mean Adequacy Ratio as an outcome: Baseline, waves 3 and 4 Healthy Aging in Neighborhoods of Diversity across the Life Span study, Table S3. Comparison of biomarkers for normal nutritional status and at-risk for malnutrition groups: Wave 4 of HANDLS study, Table S4: Predictors of risk for malnutrition determined by Mini Nutritional Assessment Score of Wave 4 HANDLS study participants.
