# Supplementary material for: A Longitudinal Assessment of Diet Quality and Risks Associated with Malnutrition in Socioeconomic and Racially Diverse Adults
Source: Nutrients. 2019 Sep 2;11(9):2046. doi: 10.3390/nu11092046 (PMC6770204; doi:10.3390/nu11092046)
Supplement: Supplementary file 1 [file nutrients-11-02046-s001.pdf]

Table 1. MAR and NAR scores of HANDLS study sample at Baseline, Wave 3 and Wave 4.

Superscripts with different letters in a row with each age group are significantly different p< 0.05. Abbreviations: HANDLS – Healthy Aging in

MAR and NAR scores, X±SE	Baseline age <50 years			Baseline age ≥50 years		
	**Baseline n=1194**	**Wave 3 n=1190**	**Wave 4 n=1198**	**Baseline n=983**	**Wave 3 n=950**	**Wave 4 n=870**
MAR	78.30±0.54[a]	77.49±0.40[a]	77.90±0.41[a]	77.68±0.60[a]	76.93±0.45[a]	74.24±0.51[b]
NAR Calcium	0.647±0.008[a]	0.735±0.007[b]	0.697±0.007[c]	0.591±0.009[a]	0.665±0.009[b]	0.539±0.011[c]
NAR Copper	0.899±0.005[a]	0.904±0.005[a]	0.879±0.005[b]	0.899±0.005[a]	0.893±0.006[a]	0.860±0.006[b]
NAR Iron	0.788±0.007[a]	0.833±0.007[b]	0.856±0.007[c]	0.941±0.005[a]	0.953±0.004[a]	0.809±0.012[b]
NAR Magnesium	0.629±0.007[a]	0.676±0.006[b]	0.669±0.007[b]	0.615±0.007[a]	0.654±0.007[b]	0.633±0.008[ab]
NAR Phosphorus	0.951±0.004[a]	0.970±0.003[b]	0.969±0.003[b]	0.948±0.004[a]	0.961±0.004[a]	0.959±0.004[a]
NAR Selenium	0.969±0.003[a]	0.977±0.003[a]	0.978±0.003[a]	0.962±0.004[a]	0.969±0.004[a]	0.970±0.003[a]
NAR Zinc	0.860±0.006[a]	0.865±0.006[a]	0.837±0.006[b]	0.837±0.007[a]	0.843±0.007[a]	0.801±0.007[b]
NAR Thiamin	0.880±0.006[a]	0.906±0.005[b]	0.893±0.005[ab]	0.863±0.006[a]	0.890±0.006[b]	0.868±0.007[a]
NAR Folate	0.833±0.007[ab]	0.851±0.006[a]	0.823±0.006[b]	0.807±0.007[a]	0.836±0.007[b]	0.795±0.008[a]
NAR Niacin	0.941±0.004[a]	0.957±0.003[b]	0.951±0.004[ab]	0.926±0.005[a]	0.938±0.005[a]	0.936±0.005[a]

Neighborhoods of Diversity across the Life Span, MAR - Mean Adequacy Ratio, NAR- Nutrient Adequacy Ratio.

NAR Riboflavin	0.938±0.004[a]	0.937±0.004[a]	0.941±0.004[a]	0.934±0.005[a]	0.936±0.005[a]	0.925±0.005[a]
NAR Vitamin A	0.794±0.040[a]	0.609±0.009[b]	0.610±0.009[b]	0.816±0.049[a]	0.641±0.009[b]	0.620±0.010[b]
NAR Vitamin B_6	0.883±0.006[a]	0.900±0.005[a]	0.881±0.006[a]	0.817±0.007[a]	0.847±0.007[b]	0.714±0.012[c]
NAR Vitamin B_{12}	0.916±0.005[a]	0.915±0.006[a]	0.905±0.006[a]	0.906±0.006[a]	0.914±0.006[ab]	0.874±0.007[c]
NAR Vitamin C	0.727±0.024[a]	0.479±0.012[b]	0.534±0.010[b]	0.692±0.025[a]	0.500±0.013[b]	0.568±0.012[c]
NAR Vitamin D	0.231±0.006[a]	0.193±0.006[b]	0.283±0.007[c]	0.250±0.007[a]	0.185±0.006[b]	0.268±0.008[a]
NAR Vitamin E	0.427±0.007[a]	0.468±0.007[b]	0.537±0.007[c]	0.401±0.008[a]	0.453±0.008[b]	0.483±0.008[c]

Table S2. Mixed effects regression with Mean Adequacy Ratio as outcome:

Baseline, Waves 3 and 4 Healthy Aging in Neighborhoods of Diversity across the Life Span study

	Coefficients	p
Intercept	6736.76	<0.001
cenAge	103.28	
Age_Groupold	582.58	<0.01
Sex Men	-361.95	<0.05
Race AA	-421.29	<0.01
PovStatBelow	-589.83	<0.001
cenEnergy	214.57	<0.001
UsedCigEver	-554.04	<0.001
EducationHS≥HS	287.22	<0.001
cenAge:Age_Groupold	-308.10	
cenAge:SexMen	-220.61	
cenAge:RaceAA	194.86	
cenAge:PovStatBelow	-165.82	
cenAge:cenEnergy	-15.29	
Age_Groupold:SexMen	-15.38	
Age_Groupold:RaceAA	367.48	
Age_Groupold:PovStatBelow	142.33	
Age_Groupold:cenEnergy	-82.07	<0.01
SexMen:RaceAA	322.74	
SexMen:PovStatBelow	510.61	<0.05
SexMen:cenEnergy	-78.22	<0.001
RaceAA:PovStatBelow	639.64	<0.01

RaceAA:cenEnergy	-44.08	<0.01
PovStatBelow:cenEnergy	-40.90	<0.05
cenAge:Age_Groupold:SexMen	154.90	
cenAge:Age_Groupold:RaceAA	-246.47	
cenAge:Age_Groupold:PovStatBelow	486.38	
cenAge:Age_Groupold:cenEnergy	76.09	<0.01
cenAge:SexMen:RaceAA	38.58	
cenAge:SexMen:PovStatBelow	28.35	
cenAge:SexMen:cenEnergy	35.12	
cenAge:RaceAA::PovStatBelow	-179.27	
cenAge:RaceAA:cenEnergy	-1.43	
cenAge:PovStatBelow:cenEnergy	-45.97	<0.05
Age_Groupold:SexMen:RaceAA	-769.92	
Age_Groupold:SexMen:PovStatBelow	-805.12	
Age_Groupold:SexMen:cenEnergy	78.64	<0.05
Age_Groupold:RaceAA:PovStatBelow	-541.75	
Age_Groupold:RaceAA:cenEnergy	178.34	<0.001
Age_Groupold:PovStatBelow:cenEnergy	181.11	<0.001
SexMen:RaceAA:PovStatBelow	-429.85	
SexMen:RaceAA:cenEnergy	41.94	<0.05
SexMen:PovStatBelow:cenEnergy	42.21	
RaceAA:PovStatBelow:cenEnergy	78.91	<0.001
cenAge:Age_Groupold:SexMen:RaceAA	0.21	
cenAge:Age_Groupold:SexMen:PovStatBelow	-109.59	
cenAge:Age_Groupold:SexMen:cenEnergy	-98.77	<0.01

cenAge:Age_Groupold:RaceAA:PovStatBelow	29.19	
cenAge:Age_Groupold:RaceAA:cenEnergy	-64.36	
cenAge:Age_Groupold:PovStatBelow:cenEnergy	-26.39	
cenAge:SexMen:RaceAA:PovStatBelow	365.06	
cenAge:SexMen:RaceAA:cenEnergy	-12.50	
cenAge:SexMen:PovStatBelow:cenEnergy	72.03	<0.05
cenAge:RaceAA:PovStatBelow:cenEnergy	65.74	<0.05
Age_Groupold:SexMen:RaceAA:PovStatBelow	1109.37	
Age_Groupold:SexMen:RaceAA:cenEnergy	-165.55	<0.001
Age_Groupold:SexMen:PovStatBelow:cenEnergy	-137.03	<0.05
Age_Groupold:RaceAA:PovStatBelow:cenEnergy	-311.46	<0.001
SexMen:RaceAA:PovStatBelow:cenEnergy	-98.59	<0.01
cenAge:Age_Groupold:SexMen:RaceAA:PovStatBelow	-351.45	
cenAge:Age_Groupold:SexMen:RaceAA:cenEnergy	118.03	<0.01
cenAge:Age_Groupold:SexMen:PovStatBelow:cenEnergy	-7.30	
cenAge:Age_Groupold:RaceAA:PovStatBelow:cenEnergy	65.43	
cenAge:SexMen:RaceAA:PovStatBelow:cenEnergy	-90.30	<0.01
Age_Groupold:SexMen:RaceAA:PovStatBelow:cenEnergy	225.44	<0.001

Abbreviations: cenAge – centered age, Age_Groupold – Age_group (≥50 years vs 30-49.9 years), SexMen – Sex(Men vs women), PovStAtBelow – Poverty status (<125% vs >125% poverty), cenEnergy – centered Energy, UsedCigEver – Cigarette smoker(yes vs no)

Table S3. Comparison of biomarkers for normal nutritional status and at-risk for malnutrition groups: Wave 4 of HANDLS study

Abbreviations: HANDLS – Healthy Aging in Neighborhoods of Diversity across the Life Span

**Biomarker**	**Normal Nutritional Status (n=435)**	**At-risk for Malnutrition (n=311)**	**p value**
WBC count (count*10^9/L), X±SE	6.38±0.10	6.67±0.13	0.071
Ferritin (ng/mL), X±SE	151.45±8.05	164.84±10.60	0.306
Platelet count (10^9/L), X±SE	236.12±3.24	238.02±4.33	0.720
Triglycerides (mg/dL), X±SE	118.62±3.10	117.00±3.44	0.728
Cholesterol [(C)/HDL-C], X±SE	3.54±0.55	3.42±0.08	0.169
Magnesium (mg/dL), X±SE	1.99±0.01	1.96±0.01	0.086

Table S4. Predictors of Risk for Malnutrition determined by Mini Nutritional Assessment Score of Wave 4 HANDLS study participants

Variables	β (SE)	p
Sex	-0.099 (0.188)	0.597
Race	-0.163 (0.178)	0.359
Poverty	-0.429 (0.187)	**0.022**
Hypertension	-0.386 (0.222)	0.082
Diabetes	-0.073 (0.099)	0.461
Cigarette smoker	-0.414 (0.118)	**0.000**
Age	0.016 (0.021)	0.460
Energy	0.000 (0.000)	**0.019**
Food insecurity	0.769 (0.221)	**0.001**
Protein, % of energy	29.166 (10.284)	**0.005**
Protein change	-11.459 (7.777)	0.141

Abbreviations: HANDLS – Healthy Aging in Neighborhoods of Diversity across the Life Span.
Bolded font was used to emphasize p -values significant at <0.05 level.
